# Depression and Artificial Intelligence Anxiety Among Chinese University Students: A Bayesian Network Analysis

**DOI:** 10.1155/da/8705382

**Published:** 2026-07-02

**Authors:** Kangyue Jin, Yuntena Wu, Tonglin Jin

**Affiliations:** ^1^ School of Psychology, Inner Mongolia Normal University, Hohhot, Inner Mongolia Autonomous Region, China, imnu.edu.cn

**Keywords:** artificial intelligence anxiety, depression, network analysis, university students

## Abstract

**Background:**

The rapid advancement of artificial intelligence (AI), while presenting opportunities for higher education, has also triggered adverse psychological consequences such as depression and AI anxiety (AIA) among Chinese university students. However, the symptom‐level associational structure and potential directional dependencies between these two constructs remain unclear.

**Methods:**

In November 2025, 1610 Chinese university students were recruited online. The Patient Health Questionnaire‐9 (PHQ‐9) and the AIA scale (AIAS) were used to measure symptoms of depression and AIA, respectively. Undirected network analysis and Bayesian network analysis were employed to explore the associations between depression and AIA symptoms.

**Results:**

Undirected network analysis revealed that PHQ4 (“Fatigue”) and PHQ6 (“Worthlessness”) were central symptoms. PHQ4 and AIA8 (“Falling behind in AI”) were identified as bridge symptoms connecting depression and AIA. Bayesian network analysis further suggested that PHQ4 is a pivotal symptom in the potential directional association between depression and AIA.

**Conclusion:**

This study provides symptom‐level evidence for the association and potential directional dependencies between depressive symptoms and AIA among Chinese university students. PHQ4 and AIA8 may play important roles in linking depressive symptoms with AIA symptoms, suggesting that they may represent potential targets for future longitudinal and intervention studies.

## 1. Introduction

Currently, the rapid development of artificial intelligence (AI) has permeated the field of higher education [1]. A survey by the Digital Education Council indicated that 86% of university students globally utilize AI for learning assistance [2]. The 55th Statistical Report on Internet Development in China further revealed that the user base for generative AI in China has reached 515 million individuals, with a high utilization rate of 47% among the higher education demographic [3]. While AI can enhance students’ learning efficiency and reduce cognitive load [4], concerns regarding AI dependence, skill depreciation, and potential job displacement have also exacerbated students’ anxiety towards AI technology and its applications [1, 5, 6].

AI anxiety (AIA) has emerged as an important concept in recent years. It refers to the fear or unease arising from individuals’ concerns about the unpredictability of AI and its potential negative consequences. AIA mainly comprises four dimensions: AI learning anxiety, which refers to concerns about learning, understanding, and mastering the functions of AI technologies; job replacement anxiety, which refers to worries that AI may replace human jobs; sociotechnical blindness, which refers to concerns about the misuse of AI and the difficulty of anticipating its social consequences; and AI configuration anxiety, which refers to the fear elicited by increasingly anthropomorphic or human‐like AI technologies [7]. Existing studies have shown that AIA is associated with adverse psychological outcomes, including burnout, fatigue, depression, reduced self‐efficacy, and lower life satisfaction [8–10]. University students are at the forefront of exploring and using generative AI. The rapid penetration of AI technologies into their learning, daily life, and career preparation may improve learning efficiency on the one hand but on the other hand, may also give rise to concerns about continuous learning pressure, weakened independent thinking, and intensified future employment competition [1, 5, 6]. Therefore, it is important to examine AIA among university students and its relationship with depressive symptoms.

In recent years, the mental health of university students has garnered considerable attention, particularly regarding their depression and anxiety [11]. A meta‐analysis encompassing 1,333,593 university students revealed a significant increase in the prevalence of anxiety and depression among Chinese university students over the past decade (2010–2020) [12]. Recent data from the Healthy Minds Network [13] also indicated a deteriorating trend in the mental health of American university students, with anxiety and depressive symptoms increasing to 34% and 38%, respectively. Concurrently, the comorbidity of anxiety and depression has been widely established across all age groups [14]. A large cohort study found that 75% of individuals with depression experienced lifetime comorbid anxiety disorders, while 81% of those with anxiety disorders experienced lifetime comorbid depression [15]. As a specific form of anxiety in the contemporary digital era, a review study found AIA to be prevalent among students [16]. However, empirical research on AIA among university students remains scarce, and the relationship between AIA and depression has not been clearly elucidated. This highlights the necessity for further research in the area of AIA among university students.

Mainstream perspectives, based on the framework of technostress theory, view AIA as an extension of technostress in the era of AI and consequently explore its relationship with individual mental health [8, 9, 17, 18]. Both constructs are rooted in adverse psychological reactions to technological development, exhibiting clear mapping relationships at the subdimensional level. For instance, AI learning anxiety corresponds to technological complexity, job displacement anxiety to technological insecurity, and sociotechnical blindness to technological uncertainty. Furthermore, AIA expands the technostress framework to include issues such as ethics and anthropomorphism. Existing research has found that the sense of loss of control brought about by the rapid iteration of AI technology, the cognitive pressure from continuously learning new technologies, and concerns about potential job displacement evoke emotional exhaustion and burnout in individuals [6, 10]. Furthermore, persistent burnout and fatigue are risk factors for inducing depression [19]. Additionally, according to the conservation of resources theory, individuals strive to acquire and accumulate resources, and when resources face actual or potential threats of loss, it triggers negative emotional reactions in individuals [20]. Liang and Zhai [5], within this theoretical framework, found that the development of AI exacerbates the psychological burden by activating perceived resource loss pathways, thereby increasing university students’ perception of employment risk. Litan’s research revealed that technostress in the age of AI is a significant predictor of depression [9].

However, according to the loss spiral principle within the conservation of resources theory, individuals experiencing psychological resource depletion or with a predisposition to depression are more prone to perceiving AI technological changes as an uncontrollable threat, thereby amplifying their perception of AIA [20]. Existing research indicates that highly neurotic individuals are more sensitive to uncertainty and a sense of loss of control and are more susceptible to experiencing anxiety in AI environments [21], whereas neuroticism is a predictor of depressive symptoms [22]. In addition, Montag et al. [23] further found that higher levels of the sadness affective system, which is closely related to depressive mood, were associated with stronger negative attitudes toward AI and greater fear of AI. Moreover, studies have identified self‐concept clarity, perceived stress, and intolerance of uncertainty as important psychological mechanisms underlying AIA [24], and these factors are likewise common cognitive and emotional vulnerabilities among individuals with depression. Notably, a longitudinal study by Huang et al. [25] found that depression predicted subsequent AI dependence, whereas AI dependence did not predict depression. Although AI dependence is not equivalent to AIA, this finding suggests that preexisting depressive symptoms may precede and exacerbate maladaptive responses to AI‐related contexts. Taken together, AIA and depression may involve a more complex bidirectional or even reverse causal relationship.

In the traditional approach, the severity of mental symptoms is typically described using total scores from standardized tools, which is widely considered to overlook the heterogeneity and potential interaction mechanisms among symptoms [26]. To address this limitation, network analysis constructs symptom networks to identify central symptoms (those most influential in maintaining the network) and bridge symptoms (those connecting different communities), thereby providing potential symptom targets for intervention [27]. However, undirected networks struggle to reveal the direction of influence among symptoms. To overcome this, some scholars have introduced directed acyclic graphs (DAGs) within Bayesian networks to further explore potential directional dependencies among symptoms [28, 29]. Therefore, this study employs a combined approach of undirected network analysis and Bayesian network analysis to comprehensively investigate the network structure and potential directional dependencies between depressive symptoms and AIA symptoms among Chinese university students, providing potential targets for future intervention strategies.

## 2. Methods

### 2.1. Study Participants

This study collected a total of 2090 questionnaires through the Credamo online data collection platform. After excluding invalid questionnaires (those that failed a lie detector question, had a completion time of less than 2 min, or exhibited regular response patterns), a total of 1610 valid questionnaires remained. The participants received compensation of 3 RMB each. Among the participants, 908 were female (56.4%) and 812 were from urban areas (50.4%). The age range of participants was 18–30 years, with a mean age of 21.69 years (SD = 1.67). Detailed demographics can be found in Table S1. This survey obtained ethical approval from the authors’ affiliated institution, and informed consent was obtained online from all participants prior to their involvement. The lie detector questions and relevant questionnaire content are provided in Table S2.

### 2.2. Measurement Tools

#### 2.2.1. AIAS

The AIAS, developed by Wang and Wang, comprises 21 items across four subdimensions [7]. AI learning anxiety consists of 8 items (e.g., “Learning to use AI technology/products makes me anxious”); job displacement anxiety consists of 6 items (e.g., “I worry that AI technology/products will replace some people’s jobs”); sociotechnical blindness consists of 4 items (e.g., “I worry that AI technology/products may be abused or misused”); and AI configuration anxiety consists of 3 items (e.g., “I find humanoid AI technology/products terrifying”). This scale uses a 7‐point Likert scale (1 = strongly disagree and 7 = strongly agree), with higher total scores indicating a greater degree of AIA. In this study, the McDonald’s ω coefficient for the total scale was 0.95, and for the factors, it ranged from 0.88 to 0.93.

#### 2.2.2. Patient Health Questionnaire‐9 (PHQ‐9)

The Patient Health Questionnaire (PHQ‐9) is a widely used self‐report scale, and its Chinese version has been validated to possess good reliability and validity [30]. It is used to assess the severity of depressive symptoms over the past 2 weeks and consists of 9 items (e.g., “Little interest or pleasure in doing things”). The scale used a 4‐point Likert scale (0 = not at all and 3 = nearly every day). Higher scores indicate more severe depressive symptoms. In this study, the McDonald’s ω coefficient for this scale was 0.87.

### 2.3. Statistical Analysis

**Figure 1:**
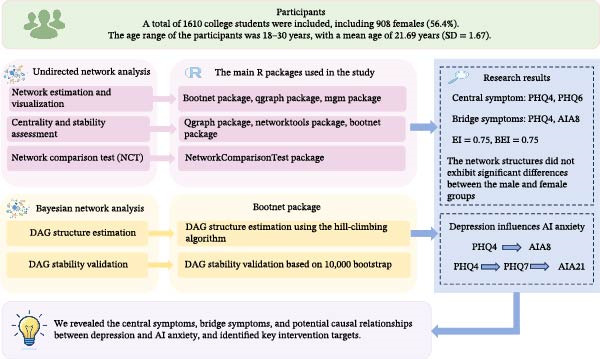
Research flow chart.

All statistical analyses were conducted using SPSS 26 and R 4.5.2. To enhance the understanding and reproducibility of the research process, we provided a research roadmap outlining the purpose of each research phase, the main R packages used in the study, and the primary research findings, as shown in Figure 1.

#### 2.3.1. Network Estimation and Visualization

This study utilized R version 4.5.2, following the methodology of Epskamp and Fried [26]. Model fitting for the data was performed using the extended Bayesian information criterion (EBIC) via the “estimateNetwork” function in the Bootnet package. The tuning parameter was set to its default value of 0.5, and the least absolute shrinkage and selection operator (LASSO) was employed to constrain the edges of the network structure, resulting in a more stringent network. The network structure was visualized using the Gaussian graphical model (GGM) from the “qgraph” package [26]. The “mgm” package was used to calculate the predictability of each node in the network. Node predictability is an indicator that assesses how well a given node is predicted by the remaining nodes in the network; a higher predictability value indicates a greater extent to which it is predicted by other symptoms in the network structure [31]. Since both depression and AIA were measured using Likert scales, polychoric coefficients were used for network analysis.

#### 2.3.2. Centrality and Stability Assessment

To quantify the importance of each node, the expected influence (EI) of each node was calculated using the “centralityPlot” function in the “qgraph” package in R software. This value represents the sum of the edge weights connecting a node to all other nodes in the network. A higher EI value indicates a greater node centrality and potential importance. Compared to traditional centrality measures (such as node strength), EI is more suitable for network structures that include both positive and negative associations [32]. The “bridge” function from the “networktools” package was used to calculate bridge expected influence (BEI) values to identify bridge symptoms between the depression community and the AIA community within the network structure [33].

To evaluate the reliability of the network analysis results, a case‐dropping bootstrap test (1000 bootstrap samples) was performed using the “bootnet” package. The correlation stability coefficient (CS‐C) was calculated to assess the stability of both centrality indices of EI and BEI. The CS‐C should not be lower than 0.25 and ideally should be above 0.50 [26]. Furthermore, the accuracy of the partial correlation coefficients was also assessed by calculating their 95% confidence intervals (CI) using a nonparametric bootstrapping test (1000 bootstrap samples).

#### 2.3.3. Network Comparison Test (NCT)

To explore potential differences in network structures between males and females, we used the “nct” function from the R package “network comparison test” [34]. We primarily conducted a network invariance test and a global strength invariance test. The network invariance test assesses whether there are significant differences between two network structures by evaluating the consistency of each edge within the networks. In contrast, the global strength invariance test examines whether the overall level of connectivity in the two networks is equal. Global strength is defined as the weighted absolute sum of all edges within a network. This test compares the global strengths of the networks to determine if there is a significant difference in their overall connectivity.

#### 2.3.4. Bayesian Network (DAG) Analysis

To address the limitation that partial correlation models may not adequately predict potential influences between nodes, we used DAGs based on Bayesian network analysis to estimate directed relationships between variables. This study used the bnlearn package in R software to compute DAGs. First, the structure of the DAG was estimated using the hill‐climbing algorithm, which iteratively adjusts by continuously adding, deleting, and reversing edges to optimize the model fit. Model fit was determined by metrics such as the Bayesian information criterion (BIC) [29]. This involved an iterative process where the procedure was randomly restarted, connecting possible edges between different node pairs, perturbing the structure, and performing 50 different random restarts to avoid local maxima. Following previous research [35], we performed 100 perturbations for each restart.

To ensure the stability of the DAG, we employed a bootstrap method (10,000 resamples with replacement) to obtain the final DAG structure, a process performed in two steps [35]. First, we counted the frequency of each edge appearing in these 10,000 bootstrapped DAGs. Following previous Bayesian network studies, we adopted a conservative edge retention threshold of 85%. Specifically, to highlight the most stable connections, improve the robustness and interpretability of the Bayesian network results, and reduce the influence of unstable or sample‐specific edges on the final network structure, an edge was considered meaningful and retained if it appeared in at least 85% of the bootstrapped networks [36]. Second, the direction of each edge was determined if, in at least 51% of the bootstrapped networks, it pointed from node X to node Y.

## 3. Results

### 3.1. Network Structure

**Figure 2:**
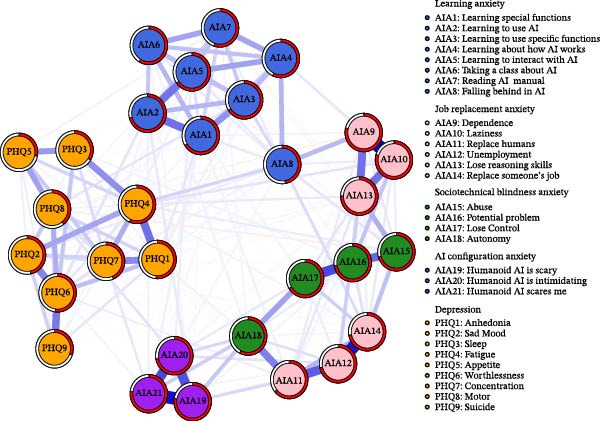
Graphical Gaussian model of the depression and AI anxiety symptom networks among university students. Edges indicate conditional dependencies or specific associations while accounting for all other variables in the network. Blue edges show positive correlations, red edges represent negative correlations, and the thickness of an edge reflects the strength of these connections, The red ring represents the predicted value.

**Figure 4:**
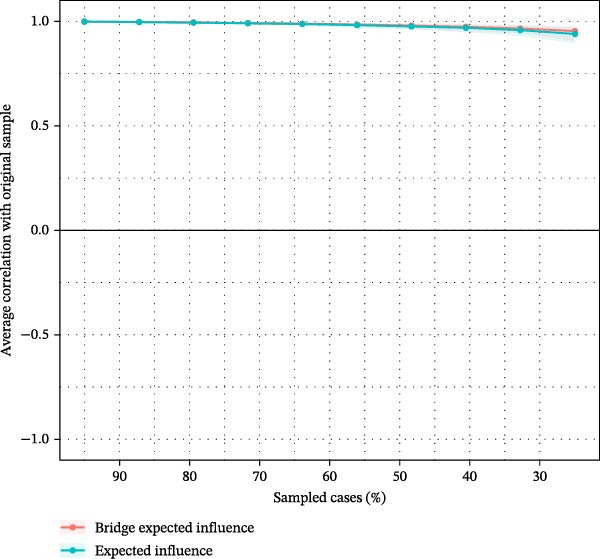
The stability of centrality and bridge centrality indices according to a case‐dropping bootstrap method.

Figure 2 displays the undirected network of depression and AIA among university students. Among them, AIA9 (“Dependence”) and AIA10 (“Laziness”) were the most closely connected, showing a strong correlation with an edge weight of 0.475. This was followed by AIA19 (“Humanoid AI is scary”) and AIA21 (“Humanoid AI scares me”), with an edge weight of 0.453 see Table S3 for details). The left panel of Figure 3 indicated that PHQ4 (“Fatigue”) and PHQ6 (“Worthlessness”) were the core symptoms with the highest EI. Their EI values were 1.843 and 1.139, respectively. The right part of Figure 3 shows that within the depression community, PHQ4 was identified as a bridge symptom, with a BEI value of 0.263. In the AIA community, AIA8 (“Falling behind in AI”) was identified as a bridge symptom, with a BEI value of 0.144. The network stability plot for the centrality indices (Figure 4) showed a relatively gradual downward trend for both EI and BEI lines. The CS‐C for both EI and BEI was 0.75, indicating ideal network stability. Figure S1 shows the 95% bootstrap CI (a small gray area), indicating high accuracy of edge weights and centrality metrics. The results of the nonparametric bootstrap difference test for centrality indices are presented in Figure S2, and the EI values and predictability values for each symptom are provided in Table S4.

Figure 3Nodal centrality index and nodal bridge centrality index of the depression and AI anxiety symptom networks among university students. (a) Nodal centrality index. (b) Nodal bridge centrality index.

**Figure:**
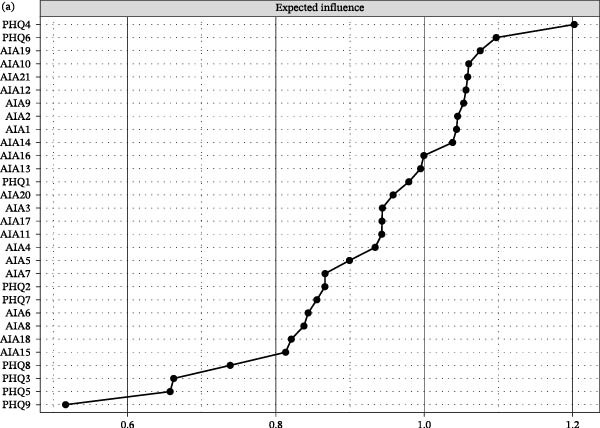

**Figure:**
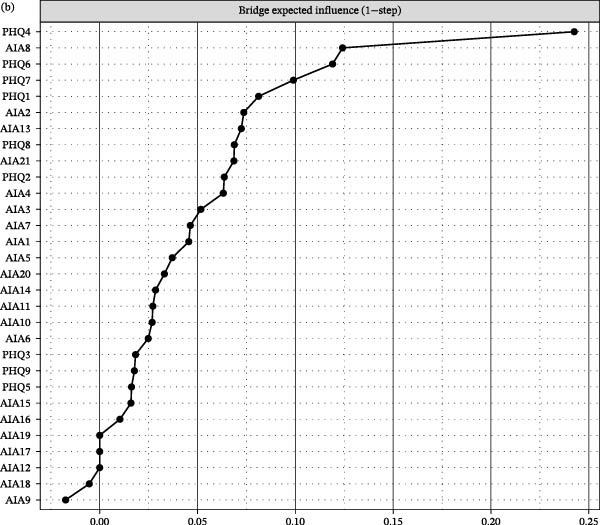

### 3.2. NCT

A comparison of the network models for 702 males (43.6%) and 908 females (56.4%) revealed no significant differences in the network structure of depression and AIA between males and females, as indicated by the overall network strength (male network strength: 14.29; female: 14.27; *S* = 0.05, *p* = 0.83) and edge weights (*M* = 0.13, *p* = 0.33).

### 3.3. Bayesian Network Structure (DAG)

**Figure 5:**
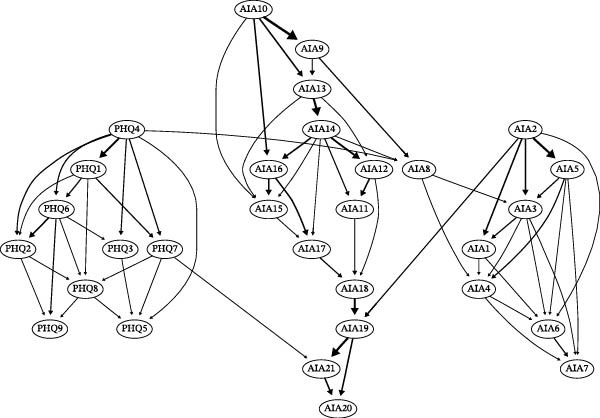
A Bayesian network (directed acyclic graph; DAG) depicting depression and AI anxiety symptoms in university students.

Figure 5 presents the DAG derived from Bayesian network analysis, which was used to explore potential directional dependencies among depressive symptoms and AIA symptoms. The DAG suggested two putative directional pathways from depressive symptoms to AIA symptoms. The first bridge‐related pathway involved a probabilistic directional link from PHQ4 (“Fatigue”) to AIA8 (“Falling behind in AI”) (direction = 0.55 and strength = 0.90). As shown in the DAG, PHQ4 occupied an upstream position within the depression symptom subnetwork and showed directed edges to PHQ1 (“Anhedonia”), PHQ2 (“Sad mood”), PHQ3 (“Sleep”), PHQ5 (“Appetite”), PHQ6 (“Worthlessness”), and PHQ7 (“Concentration”). PHQ4 was also probabilistically directed toward AIA8, and AIA8 further showed directed connections with other AIA symptoms, suggesting a possible pathway through which fatigue may be linked to broader AIA symptoms. The second potential pathway involved a probabilistic directional link from PHQ7 (“Concentration”) to AIA21 (“Humanoid AI scares me”) (direction = 0.63 and strength = 0.96). AIA21 further showed directed connections with other AIA symptoms, suggesting another possible pathway through which concentration problems may be linked to AI configuration‐related anxiety symptoms. It is noteworthy that the most influential activating symptom and bridge symptom in the DAG are PHQ4 and AIA8, both located upstream in the network. This finding is consistent with the results from the undirected network analysis, indicating that PHQ4 and AIA8 may be key symptoms that activate and maintain both AIA and depressive symptoms, making them potential intervention targets for the future. The strength and directional probability results between nodes in the bootstrapped network are provided in Table S5.

## 4. Discussion

This study comprehensively utilized undirected network analysis and Bayesian network analysis to explore the complex associations and potential directional dependencies between depressive symptoms and AIA symptoms among Chinese university students. Specifically, the undirected network revealed the central and bridge symptoms within the depression and AIA networks. Building upon this, the Bayesian network further elucidated the potential directional dependencies and influence pathways among these symptoms.

### 4.1. Regularized Partial Correlation Network

In the undirected network model, PHQ4 (“Fatigue”) was identified as the most central symptom and bridge symptom in the depression and AIA network, followed by PHQ6 (“Worthlessness”). Consistent with previous research on comorbidity between depression and anxiety, multiple studies have found PHQ4 to be the central symptom with the highest EI in the network structure of depression and anxiety among university students [37, 38]. Furthermore, Li et al. [11] utilized large‐sample data across three time points to investigate the relationships among depression, anxiety, and insomnia symptoms in Chinese university students during COVID‐19 and found PHQ4 to be a central and bridge symptom in all three study phases. The convergent findings from these studies indicate that fatigue plays a significant role in the comorbidity symptom network of depression and anxiety among university students. This study reveals that even though AIA is a novel manifestation of anxiety, fatigue also dominates the comorbidity link between depression and AIA. Therefore, fatigue among university students is a public health issue that warrants significant attention.

Fatigue is characterized by prolonged feelings of tiredness and a lack of energy [39, 40]. Previous research has found that fatigue is highly associated with depression, with 41.2% of patients with major depressive disorder exhibiting fatigue symptoms and 30.4% experiencing severe fatigue symptoms [19]. University students are at a critical stage of academic and career development and face multiple stressors, such as academic competition, job uncertainty, and the rapid iteration of AI technologies [5, 6]. These stressors may continuously deplete individuals’ cognitive and emotional resources, thereby contributing to fatigue and negative emotions such as depression [10, 39, 41]. Furthermore, fatigue can significantly impair individuals’ cognitive and executive functions [39, 42]. The depletion of cognitive resources may make it difficult for students to rationally evaluate the complexity and uncertainty of AI technologies, thereby amplifying anxiety and fear of AI [43]. The undirected network analysis in this study suggests that fatigue may be a promising symptom‐level target for future interventions aimed at reducing comorbid depressive symptoms and AIA.

In the undirected network model, PHQ6 (“Worthlessness”) also exhibited high centrality, consistent with previous findings that worthlessness is a central symptom in depression–anxiety comorbidity networks among adolescents [44]. This suggests that PHQ6 may be a particularly relevant central symptom in younger populations. Worthlessness may be closely related to low self‐efficacy, which has been associated with perceived stress and depressive symptoms [45, 46]. In the context of AI, the rapid development of AI technology may increase students’ uncertainty about future career development and contribute to stress related to academic and occupational competence [5, 47]. According to the Social Cognitive Theory, self‐efficacy is a crucial factor enabling individuals to cope with challenges and uncertainties, and individuals with low self‐efficacy tend to underestimate their ability to cope with threatening or uncertain situations [48, 49]. Thus, students with lower self‐efficacy may be more prone to feelings of loss of control when facing the complexity and uncertainty of AI technology, which may, in turn, be associated with higher AIA [50]. Therefore, PHQ6 may represent a potential symptom‐level target for future interventions addressing comorbid depressive symptoms and AIA.

In the undirected network model, PHQ4 (“Fatigue”) and AIA8 (“Falling behind in AI”) emerged as key bridge symptoms, acting as critical nodes connecting depressive symptoms and AIA symptoms. The significant bridging effect of PHQ4 within depressive symptoms suggests that feeling tired or having little energy might be a crucial channel that potentially activates or exacerbates AIA among university students. Among AIA symptoms, AIA8 exhibited the strongest bridging effect. This reflects that AI‐related learning anxiety may induce depressive symptoms by increasing students’ academic pressure and psychological burden [6, 51]. The comorbidity between AIA and depression might not be a generalized association but rather a mutual influence and maintenance through specific, measurable symptom channels. Interventions targeting these bridge symptoms might block the development of comorbid depression and AIA among university students.

### 4.2. Bayesian Network

To address the limitation that undirected networks primarily reveal conditional associations among symptoms, this study further employed a Bayesian network DAG to explore potential directional dependencies among symptoms. The DAG suggested that depressive symptoms may show putative directional links to AIA symptoms. A key bridge‐related pathway involved PHQ4 (“Fatigue”) as a potentially upstream depressive symptom that was probabilistically directed toward AIA8 (“Falling behind in AI”), which was further connected with other AIA symptoms. This finding provides a new perspective for understanding the relationship between depression and AIA. Mainstream views often consider the development of AI technology as an external stressor, subsequently investigating its impact on individual mental health [8, 9, 18]. However, the findings of this study suggest that depressive symptoms may be a risk factor influencing AIA, aligning with the loss spiral principle in the conservation of Resources Theory [20].

The second putative directional pathway involved PHQ4 (“Fatigue”) as an upstream symptom that was linked to PHQ7 (“Concentration”), which was further probabilistically directed toward AIA21 (“Humanoid AI scares me”). Current research on anthropomorphic AI presents seemingly contradictory views: on the one hand, the uncanny valley theory suggests that highly anthropomorphic AI may evoke human fear and revulsion [52]; on the other hand, studies also find that anthropomorphic AI designs can enhance human trust [53]. This study finds that different attitudes towards humanoid AI in AI configurations may depend on individual cognitive differences. Difficulty concentrating is a manifestation of cognitive impairment in depressive symptoms [39]. Fatigue depletes limited attentional control resources, and the exhaustion of cognitive resources may cause individuals to amplify their perception of threatening events [43, 54]. Therefore, individuals in a state of cognitive resource depletion may be more prone to triggering the uncanny valley effect, leading to anxiety and avoidance of humanoid AI.

## 5. Research Limitations and Future Prospects

This study has several limitations. First, the sample consisted solely of university students, and symptom network structures may vary across populations. Therefore, future research should extend this work to other populations to enhance the generalizability of the findings. Second, the current network did not control for potential confounders, such as emotion regulation strategies and personality traits, which are closely associated with anxiety and depression. Future studies should account for these factors to further examine the robustness of the network structure. Finally, this study used a cross‐sectional design. Although Bayesian network analysis can suggest potential directional dependencies between symptoms through conditional probabilities, it does not establish the temporal ordering of symptoms. Therefore, longitudinal studies are needed in the future.

## 6. Conclusion

This study combined undirected network analysis with a Bayesian DAG model to explore symptom‐level associations and potential directional dependencies between depressive symptoms and AIA among Chinese university students. The undirected network analysis identified PHQ4 (“Fatigue”) and PHQ6 (“Worthlessness”) as central symptoms, while PHQ4 and AIA8 (“Falling behind in AI”) emerged as bridge symptoms linking depressive symptoms with AIA symptoms. In the Bayesian DAG, PHQ4 occupied a putative upstream position within the depression symptom subnetwork and showed a probabilistic directional link with AIA8. In addition, PHQ7 (“Concentration”) showed a probabilistic directional link with AIA21 (“Humanoid AI scares me”), suggesting a possible pathway from depressive symptoms to AIA symptoms. Given the cross‐sectional design of this study, the DAG‐based directions should be interpreted as statistical directional dependencies rather than evidence of temporal or causal ordering. Overall, these findings provide symptom‐level evidence for the association between depression and AIA and suggest potential targets for future longitudinal and intervention studies.

## Author Contributions

**Kangyue Jin**: methodology, software, formal analysis, data curation, visualization, writing – original draft. **Tonglin Jin**: conceptualization, writing – review and editing. **Yuntena Wu**: conceptualization, project administration, writing – review and editing.

## Funding

This research was funded by the National Social Science Fund Project (Grant 23BSH142).

## Conflicts of Interest

The authors declare no conflicts of interest.

## Supporting Information

Additional supporting information can be found online in the Supporting Information section.

## Supporting information


**Supporting Information** Table S1: Sociodemographic characteristics of the participants. Table S2: Items of the Artificial Intelligence Anxiety Scale and the Patient Health Questionnaire‐9. Table S3: Correlation matrix of the depression and AI anxiety symptom network. Figure S1: Accuracy estimation of network edge weights for the depression and AI anxiety symptom networks among college students. Figure S2: Nonparametric bootstrapping difference test for centrality indices. Table S4: Network scores for depression and AI anxiety symptoms. Table S5: Edge strength and direction probability between depression and AI anxiety nodes in the Bayesian network.

## Data Availability

The data will be made available upon request.
