# Supplementary material for: Depression and Artificial Intelligence Anxiety Among Chinese University Students: A Bayesian Network Analysis
Source: Depress Anxiety. 2026 Jul 2;2026:8705382. doi: 10.1155/da/8705382 (PMC13329113; doi:10.1155/da/8705382)
Supplement: Supplementary file 1 — Supporting Information Table S1: Sociodemographic characteristics of the participants. Table S2: Items of the Artificial Intelligence Anxiety Scale and the Patient Health Questionnaire‐9. Table S3: Correlation matrix of the depression and AI anxiety symptom network. Figure S1: Accuracy estimation of network edge weights for the depression and AI anxiety symptom networks among college students. Figure S2: Nonparametric bootstrapping difference test for centrality indices. Table S4: Network scores for depression and AI anxiety symptoms. Table S5: Edge strength and direction probability between depression and AI anxiety nodes in the Bayesian network. [file DA-2026-8705382-s001.docx]

**Supplementary Materials**

Table S1. Sociodemographic data.

Table S2. Artiﬁcial Intelligence Anxiety Scale

Patient Health Questionnaire-9

Table S3. Correlation matrix of the Depression and AI Anxiety Symptom Network

Table S4. Depression and AI Anxiety Symptom Networks Scores

Table S5. Edge strength and direction probability between the Depression and AI Anxiety nodes in a Bayesian network.

Figure S1. Accuracy estimation of network edge weights of the Depression and AI Anxiety Symptom Networks Among College Students

Figure S2. Non-parametric bootstrapping difference test for centrality indices

Table S1

Sociodemographic data.

|  | M(SD)/N(%) |
| --- | --- |
| Age | 21.69(1.67) |
| Gender |  |
| Male | 702(43.6%) |
| Female | 908(56.4%) |
| Location |  |
| Urban | 812(50.4%) |
| Rural | 798(49.6%) |
| Academic year |  |
| Freshman | 34(2.1%) |
| Sophomore | 234(14.5%) |
| Junior | 501(31.1%) |
| Senior | 559(34.7%) |
| Graduate | 272(16.9%) |
| PhD | 10(0.6%) |
| Frequency of AI Use |  |
| Daily use | 1056(65.6%) |
| 2-3 times a week | 536(33.3%) |
| Once a week or less | 18(1.1%) |
| Major |  |
| STEM | 669(41.6%) |
| Humanities and Social Sciences | 375(23.3%) |
| Arts and Design | 37(2.3%) |
| Business and Economics | 391(24.3%) |
| Medical Sciences | 138(8.6%) |

Table S2

Artiﬁcial Intelligence Anxiety Scale

| 1. Learning to understand all of the special functions associated with an AI technique/product makes me anxious. | 1 | 2 | 3 | 4 | 5 | 6 | 7 |
| --- | --- | --- | --- | --- | --- | --- | --- |
| 2. Learning to use AI techniques/products makes me anxious. | 1 | 2 | 3 | 4 | 5 | 6 | 7 |
| 3. Learning to use speciﬁc functions of an AI technique/product makes me  anxious. | 1 | 2 | 3 | 4 | 5 | 6 | 7 |
| 4. Learning how an AI technique/product works makes me anxious. | 1 | 2 | 3 | 4 | 5 | 6 | 7 |
| 5. Learning to interact with an AI technique/product makes me anxious. | 1 | 2 | 3 | 4 | 5 | 6 | 7 |
| 6. Taking a class about the development of AI techniques/products makes me anxious. | 1 | 2 | 3 | 4 | 5 | 6 | 7 |
| Q1. This is a screening question. Please select the option that represents somewhat agreeing. | 1 | 2 | 3 | 4 | 5 | 6 | 7 |
| 7. Reading an AI technique/product manual makes me anxious. | 1 | 2 | 3 | 4 | 5 | 6 | 7 |
| 8. Being unable to keep up with the advances associated with AI techniques/products makes me anxious. | 1 | 2 | 3 | 4 | 5 | 6 | 7 |
| 9. I am afraid that an AI technique/product may make us dependent. | 1 | 2 | 3 | 4 | 5 | 6 | 7 |
| 10. I am afraid that an AI technique/product may make us even lazier. | 1 | 2 | 3 | 4 | 5 | 6 | 7 |
| 11. I am afraid that an AI technique/product may replace humans. | 1 | 2 | 3 | 4 | 5 | 6 | 7 |
| 12. I am afraid that widespread use of humanoid robots will take jobs away from people. | 1 | 2 | 3 | 4 | 5 | 6 | 7 |
| 13. I am afraid that if I begin to use AI techniques/products I will become dependent upon them and lose some of my reasoning skills. | 1 | 2 | 3 | 4 | 5 | 6 | 7 |
| 14. I am afraid that AI techniques/products will replace someone’s job. | 1 | 2 | 3 | 4 | 5 | 6 | 7 |
| 15. I am afraid that an AI technique/product may be misused. | 1 | 2 | 3 | 4 | 5 | 6 | 7 |
| 16. I am afraid of various problems potentially associated with an AI technique/product. | 1 | 2 | 3 | 4 | 5 | 6 | 7 |
| 17. I am afraid that an AI technique/product may get out of control and  malfunction. | 1 | 2 | 3 | 4 | 5 | 6 | 7 |
| 18. I am afraid that an AI technique/product may lead to robot autonomy. | 1 | 2 | 3 | 4 | 5 | 6 | 7 |
| 19. I ﬁnd humanoid AI techniques/products (e.g. humanoid robots) scary. | 1 | 2 | 3 | 4 | 5 | 6 | 7 |
| 20. I ﬁnd humanoid AI techniques/products (e.g. humanoid robots)  intimidating. | 1 | 2 | 3 | 4 | 5 | 6 | 7 |
| 21. I don’t know why, but humanoid AI techniques/products (e.g. humanoid robots) scare me. | 1 | 2 | 3 | 4 | 5 | 6 | 7 |

Patient Health Questionnaire-9

| 1. Little interest or pleasure in doing things | 0 | 1 | 2 | 3 |
| --- | --- | --- | --- | --- |
| 2. Feeling down, depressed, or hopeless | 0 | 1 | 2 | 3 |
| 3. Trouble falling or staying asleep, or sleeping too much | 0 | 1 | 2 | 3 |
| 4. Feeling tired or having little energy | 0 | 1 | 2 | 3 |
| 5. Poor appetite or overeating | 0 | 1 | 2 | 3 |
| 6. Feeling bad about yourself — or that you are a failure or have let yourself or your family down | 0 | 1 | 2 | 3 |
| 7. Trouble concentrating on things, such as reading the newspaper or watching television | 0 | 1 | 2 | 3 |
| 8. Moving or speaking so slowly that other people could have noticed? Or the opposite — being so fidgety or restless that you have been moving around a lot more than usual | 0 | 1 | 2 | 3 |
| 9. Thoughts that you would be better off dead or of hurting yourself in some way | 0 | 1 | 2 | 3 |

Table S3 Correlation matrix of the Depression and AI Anxiety Symptom Network

|  | 1 | 2 | 3 | 4 | 5 | 6 | 7 | 8 | 9 | 10 | 11 | 12 | 13 | 14 | 15 | 16 | 17 | 18 | 19 | 20 | 21 | 22 | 23 | 24 | 25 | 26 | 27 | 28 | 29 | 30 |
| --- | --- | --- | --- | --- | --- | --- | --- | --- | --- | --- | --- | --- | --- | --- | --- | --- | --- | --- | --- | --- | --- | --- | --- | --- | --- | --- | --- | --- | --- | --- |
| 1 AIA1 | 0 |  |  |  |  |  |  |  |  |  |  |  |  |  |  |  |  |  |  |  |  |  |  |  |  |  |  |  |  |  |
| 2 AIA2 | 0.267 | 0 |  |  |  |  |  |  |  |  |  |  |  |  |  |  |  |  |  |  |  |  |  |  |  |  |  |  |  |  |
| 3 AIA3 | 0.200 | 0.137 | 0 |  |  |  |  |  |  |  |  |  |  |  |  |  |  |  |  |  |  |  |  |  |  |  |  |  |  |  |
| 4 AIA4 | 0.102 | 0 | 0.127 | 0 |  |  |  |  |  |  |  |  |  |  |  |  |  |  |  |  |  |  |  |  |  |  |  |  |  |  |
| 5 AIA5 | 0.062 | 0.258 | 0 | 0.108 | 0 |  |  |  |  |  |  |  |  |  |  |  |  |  |  |  |  |  |  |  |  |  |  |  |  |  |
| 6 AIA6 | 0.136 | 0.107 | 0.108 | 0.173 | 0.100 | 0 |  |  |  |  |  |  |  |  |  |  |  |  |  |  |  |  |  |  |  |  |  |  |  |  |
| 7 AIA7 | 0.084 | 0.097 | 0.122 | 0.117 | 0.114 | 0.169 | 0 |  |  |  |  |  |  |  |  |  |  |  |  |  |  |  |  |  |  |  |  |  |  |  |
| 8 AIA8 | 0.039 | 0.020 | 0.100 | 0.171 | 0.041 | 0.037 | 0.035 | 0 |  |  |  |  |  |  |  |  |  |  |  |  |  |  |  |  |  |  |  |  |  |  |
| 9 AIA9 | 0.022 | 0 | 0.070 | 0.139 | 0 | 0 | 0 | 0.131 | 0 |  |  |  |  |  |  |  |  |  |  |  |  |  |  |  |  |  |  |  |  |  |
| 10 AIA10 | 0 | 0 | 0 | 0.047 | 0 | 0 | 0 | 0.034 | 0.475 | 0 |  |  |  |  |  |  |  |  |  |  |  |  |  |  |  |  |  |  |  |  |
| 11 AIA11 | 0.027 | 0 | 0 | 0 | 0 | 0 | 0.003 | 0 | 0.036 | 0.024 | 0 |  |  |  |  |  |  |  |  |  |  |  |  |  |  |  |  |  |  |  |
| 12 AIA12 | 0 | 0 | 0 | 0 | -0.006 | 0 | 0 | 0 | 0 | 0.013 | 0.303 | 0 |  |  |  |  |  |  |  |  |  |  |  |  |  |  |  |  |  |  |
| 13 AIA13 | 0 | 0 | 0 | 0 | 0 | -0.011 | -0.013 | 0 | 0.266 | 0.262 | 0.044 | 0.091 | 0 |  |  |  |  |  |  |  |  |  |  |  |  |  |  |  |  |  |
| 14 AIA14 | 0 | 0 | 0 | 0 | -0.002 | 0 | 0 | 0.066 | 0.027 | 0.042 | 0.143 | 0.437 | 0.014 | 0 |  |  |  |  |  |  |  |  |  |  |  |  |  |  |  |  |
| 15 AIA15 | 0 | -0.003 | -0.009 | 0 | -0.018 | 0 | -0.018 | 0.048 | 0.040 | 0.036 | 0 | 0.007 | 0.148 | 0.118 | 0 |  |  |  |  |  |  |  |  |  |  |  |  |  |  |  |
| 16 AIA16 | 0 | 0 | 0 | 0 | 0 | 0 | 0 | 0.013 | 0.020 | 0.110 | 0.013 | 0.021 | 0.062 | 0.060 | 0.281 | 0 |  |  |  |  |  |  |  |  |  |  |  |  |  |  |
| 17 AIA17 | 0.021 | 0 | 0 | 0 | 0 | 0 | 0 | 0 | 0.008 | 0.012 | 0.009 | 0.032 | 0.048 | 0.086 | 0.175 | 0.341 | 0 |  |  |  |  |  |  |  |  |  |  |  |  |  |
| 18 AIA18 | 0 | 0 | 0 | 0 | 0 | 0.025 | 0.019 | 0 | 0 | 0 | 0.239 | 0.057 | 0 | 0.016 | 0 | 0. | 0.175 | 0 |  |  |  |  |  |  |  |  |  |  |  |  |
| 19 AIA19 | 0.001 | 0.042 | 0 | 0 | 0.003 | 0.021 | 0 | 0 | 0 | 0 | 0.071 | 0.022 | 0 | 0 | 0 | 0.039 | 0 | 0.105 | 0 |  |  |  |  |  |  |  |  |  |  |  |
| 20 AIA20 | 0 | 0 | 0 | 0 | 0.033 | 0.005 | 0 | 0.019 | 0.001 | 0.001 | 0 | 0.054 | 0.010 | 0 | 0 | 0.021 | 0.041 | 0.071 | 0.326 | 0 |  |  |  |  |  |  |  |  |  |  |
| 21 AIA21 | 0.015 | 0.027 | 0.013 | 0 | 0.004 | 0 | 0.027 | 0 | 0 | 0 | 0 | 0.018 | 0 | 0 | 0 | 0.001 | 0 | 0.092 | 0.453 | 0.339 | 0 |  |  |  |  |  |  |  |  |  |
| 22 PHQ1 | 0.044 | 0.011 | 0.028 | 0.021 | 0 | 0 | 0 | 0.019 | 0 | 0 | 0 | 0 | 0.013 | 0 | 0 | 0 | 0 | -0.027 | 0 | 0 | 0 | 0 |  |  |  |  |  |  |  |  |
| 23 PHQ2 | 0 | 0 | 0 | 0.017 | 0 | 0 | 0 | 0.033 | 0 | 0 | 0 | 0.007 | 0.007 | 0.010 | 0 | 0 | 0 | 0 | 0.002 | 0 | 0.019 | 0.084 | 0 |  |  |  |  |  |  |  |
| 24 PHQ3 | 0 | 0 | 0 | 0 | 0 | 0 | 0 | 0.003 | 0 | 0 | 0 | 0 | 0 | 0 | 0.001 | 0.023 | 0 | 0 | 0 | 0 | 0 | 0.026 | 0 | 0 |  |  |  |  |  |  |
| 25 PHQ4 | 0.005 | 0 | 0 | 0.017 | 0 | 0.015 | 0 | 0.079 | 0 | 0.051 | 0 | 0 | 0.039 | 0.022 | 0.036 | 0 | 0 | 0 | 0 | 0 | 0 | 0.266 | 0.167 | 0.206 | 0 |  |  |  |  |  |
| 26 PHQ5 | 0 | 0.008 | 0 | 0 | 0 | 0 | 0.025 | 0 | 0 | 0 | 0 | 0 | 0 | 0 | 0 | 0 | 0 | 0 | 0 | 0 | 0 | 0.019 | 0.028 | 0.189 | 0.078 | 0 |  |  |  |  |
| 27 PHQ6 | 0 | 0.050 | 0 | 0 | 0.007 | 0 | 0.011 | 0.024 | 0 | 0 | 0.031 | 0.001 | 0 | 0 | 0 | 0 | 0 | 0 | 0 | 0 | 0.004 | 0.121 | 0.230 | 0.108 | 0.046 | 0.008 | 0 |  |  |  |
| 28 PHQ7 | 0 | 0.023 | 0 | 0 | 0.002 | 0 | 0.027 | 0 | 0 | 0.005 | 0 | 0 | 0.015 | 0 | 0 | -0.005 | 0 | 0 | 0 | 0 | 0.046 | 0.222 | 0.066 | 0.066 | 0.154 | 0.098 | 0.055 | 0 |  |  |
| 29 PHQ8 | 0.008 | 0.001 | 0.047 | 0 | 0 | 0 | 0.013 | -0.015 | -0.021 | 0 | 0 | 0 | 0 | 0 | -0.029 | 0 | 0 | 0.009 | 0 | 0.051 | 0 | 0.083 | 0.106 | 0.022 | 0.022 | 0.174 | 0.152 | 0.058 | 0 |  |
| 30 PHQ9 | 0 | 0 | 0 | 0.002 | 0.017 | 0 | 0 | 0 | 0 | -0.011 | 0 | 0 | 0 | 0 | 0 | 0 | -0.004 | 0 | 0 | 0 | 0 | 0.049 | 0.089 | 0.017 | 0 | 0.30 | 0.248 | 0.023 | 0.055 | 0 |

Table S4 Depression and AI Anxiety Symptom Networks Scores（*n*＝1610）

| Item | Item Content | Mean(SD） | EI | BEI | Predictability |
| --- | --- | --- | --- | --- | --- |
| AIA1 | Learning special functions | 2.84(1.27) | 0.781 | 0.057 | 0.664 |
| AIA2 | Learning to use AI | 2.39(1.35) | 0.791 | 0.092 | 0.711 |
| AIA3 | Learning to use specific functions | 2.60(1.34) | 0.113 | 0.075 | 0.660 |
| AIA4 | Learning about how AI works | 2.98(1.51) | 0.048 | 0.057 | 0.573 |
| AIA5 | Learning to interact with AI | 2.57(1.33) | -0.184 | 0.026 | 0.651 |
| AIA6 | Taking a class about AI | 2.55(1.44) | -0.557 | 0.015 | 0.588 |
| AIA7 | Reading AI manual | 2.62(1.36) | -0.404 | 0.076 | 0.555 |
| AIA8 | Falling behind in AI | 3.81(1.73) | -0.595 | 0.144 | 0.474 |
| AIA9 | Dependence | 4.71(1.61) | 0.846 | -0.021 | 0.789 |
| AIA10 | Laziness | 4.75(1.71) | 0.892 | 0.044 | 0.796 |
| AIA11 | Replace humans | 3.51(1.68) | 0.107 | 0.031 | 0.642 |
| AIA12 | Unemployment | 4.14(1.75) | 0.867 | 0.008 | 0.717 |
| AIA13 | Lose reasoning skills | 4.68(1.70) | 0.458 | 0.074 | 0.753 |
| AIA14 | Replace someone’s job | 4.44(1.70) | 0.746 | 0.032 | 0.723 |
| AIA15 | Abuse | 4.95(1.72) | -0.760 | 0.007 | 0.706 |
| AIA16 | Potential problem | 4.56(1.64) | 0.487 | 0.017 | 0.723 |
| AIA17 | Lose control | 4.33(1.63) | 0.110 | -0.004 | 0.664 |
| AIA18 | Autonomy | 3.31(1.67) | -0.708 | -0.018 | 0.576 |
| AIA19 | Humanoid AI is scary | 3.08(1.63) | 0.996 | 0.002 | 0.778 |
| AIA20 | Humanoid AI is intimidating | 3.22(1.60) | 0.210 | 0.051 | 0.723 |
| AIA21 | Humanoid AI scares me | 2.95(1.70) | 0.882 | 0.069 | 0.771 |
| PHQ1 | Anhedonia | 0.91(0.76) | 0.351 | 0.108 | 0.516 |
| PHQ2 | Sad Mood | 0.86(0.77) | -0.406 | 0.095 | 0.468 |
| PHQ3 | Sleep | 1.06(0.94) | -1.769 | 0.027 | 0.338 |
| PHQ4 | Fatigue | 1.36(0.90) | 1.843 | 0.263 | 0.566 |
| PHQ5 | Appetite | 0.80(0.86) | -1.802 | 0.033 | 0.283 |
| PHQ6 | Worthlessness | 0.70(0.86) | 1.139 | 0.127 | 0.520 |
| PHQ7 | Concentration | 0.92(0.85) | -0.478 | 0.113 | 0.436 |
| PHQ8 | Motor | 0.57(0.74) | -1.260 | 0.065 | 0.393 |
| PHQ9 | Suicide | 0.21(0.51) | -2.744 | 0.004 | 0.303 |

Table S5 Edge strength and direction probability between the Depression and AI Anxiety nodes in a Bayesian network.

| **from** | **to** | **strength** | **direction** |
| --- | --- | --- | --- |
| PHQ1 | PHQ2 | 0.992 | 0.612 |
| PHQ1 | PHQ6 | 1 | 0.578 |
| PHQ1 | PHQ7 | 1 | 0.777 |
| PHQ1 | PHQ8 | 0.931 | 0.867 |
| PHQ2 | PHQ8 | 0.994 | 0.788 |
| PHQ2 | PHQ9 | 0.999 | 0.837 |
| PHQ3 | PHQ5 | 1 | 0.731 |
| PHQ4 | PHQ1 | 1 | 0.574 |
| PHQ4 | PHQ2 | 1 | 0.727 |
| PHQ4 | PHQ3 | 1 | 0.908 |
| PHQ4 | PHQ5 | 0.855 | 0.97 |
| PHQ4 | PHQ6 | 0.883 | 0.656 |
| PHQ4 | PHQ7 | 1 | 0.791 |
| PHQ4 | AIA8 | 0.9 | 0.549 |
| PHQ6 | PHQ2 | 1 | 0.545 |
| PHQ6 | PHQ3 | 0.995 | 0.772 |
| PHQ6 | PHQ8 | 1 | 0.852 |
| PHQ6 | PHQ9 | 1 | 0.915 |
| PHQ7 | PHQ5 | 0.994 | 0.921 |
| PHQ7 | PHQ8 | 0.982 | 0.696 |
| PHQ7 | AIA21 | 0.955 | 0.629 |
| PHQ8 | PHQ5 | 0.999 | 0.847 |
| PHQ8 | PHQ9 | 0.885 | 0.732 |
| AIA1 | AIA4 | 0.993 | 0.563 |
| AIA1 | AIA6 | 0.993 | 0.657 |
| AIA2 | AIA1 | 1 | 0.673 |
| AIA2 | AIA3 | 1 | 0.754 |
| AIA2 | AIA5 | 1 | 0.72 |
| AIA2 | AIA6 | 0.962 | 0.861 |
| AIA2 | AIA19 | 0.926 | 0.553 |
| AIA3 | AIA1 | 1 | 0.52 |
| AIA3 | AIA4 | 0.999 | 0.575 |
| AIA3 | AIA6 | 0.99 | 0.757 |
| AIA3 | AIA7 | 0.905 | 0.843 |
| AIA4 | AIA6 | 0.991 | 0.729 |
| AIA4 | AIA7 | 1 | 0.843 |
| AIA5 | AIA3 | 0.998 | 0.51 |
| AIA5 | AIA4 | 1 | 0.623 |
| AIA5 | AIA6 | 0.997 | 0.68 |
| AIA5 | AIA7 | 0.922 | 0.794 |
| AIA6 | AIA7 | 1 | 0.706 |
| AIA8 | AIA3 | 0.981 | 0.764 |
| AIA8 | AIA4 | 0.998 | 0.753 |
| AIA9 | AIA8 | 0.976 | 0.814 |
| AIA9 | AIA13 | 1 | 0.572 |
| AIA10 | AIA9 | 1 | 0.509 |
| AIA10 | AIA13 | 1 | 0.582 |
| AIA10 | AIA15 | 0.859 | 0.671 |
| AIA10 | AIA16 | 0.981 | 0.65 |
| AIA11 | AIA18 | 1 | 0.781 |
| AIA12 | AIA11 | 1 | 0.784 |
| AIA12 | AIA18 | 0.95 | 0.878 |
| AIA13 | AIA12 | 0.862 | 0.608 |
| AIA13 | AIA14 | 0.898 | 0.579 |
| AIA13 | AIA15 | 1 | 0.643 |
| AIA14 | AIA8 | 0.935 | 0.734 |
| AIA14 | AIA11 | 0.999 | 0.756 |
| AIA14 | AIA12 | 1 | 0.624 |
| AIA14 | AIA15 | 0.999 | 0.502 |
| AIA14 | AIA16 | 0.878 | 0.534 |
| AIA14 | AIA17 | 0.86 | 0.714 |
| AIA15 | AIA17 | 1 | 0.794 |
| AIA16 | AIA15 | 1 | 0.512 |
| AIA16 | AIA17 | 1 | 0.809 |
| AIA17 | AIA18 | 1 | 0.692 |
| AIA18 | AIA19 | 0.981 | 0.924 |
| AIA19 | AIA20 | 1 | 0.692 |
| AIA19 | AIA21 | 1 | 0.525 |
| AIA21 | AIA20 | 1 | 0.647 |

**
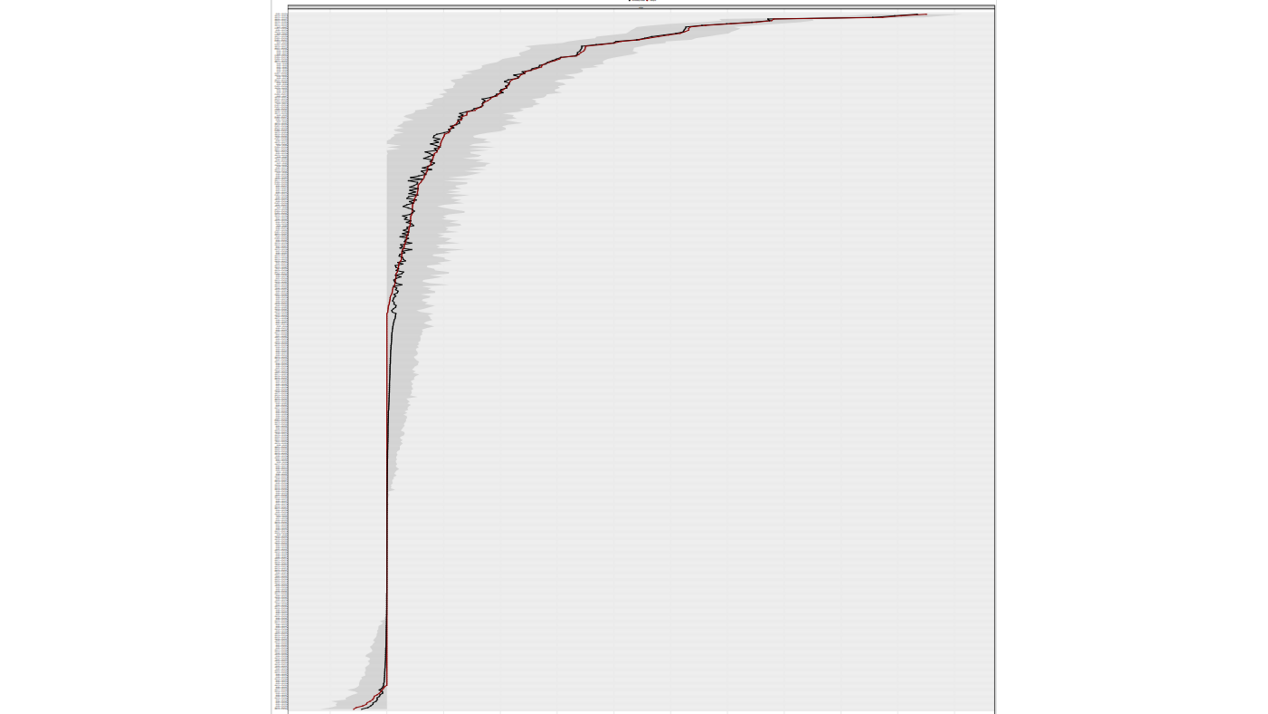
**

Figure S1. Accuracy estimation of network edge weights of the Depression and AI Anxiety Symptom Networks Among College Students


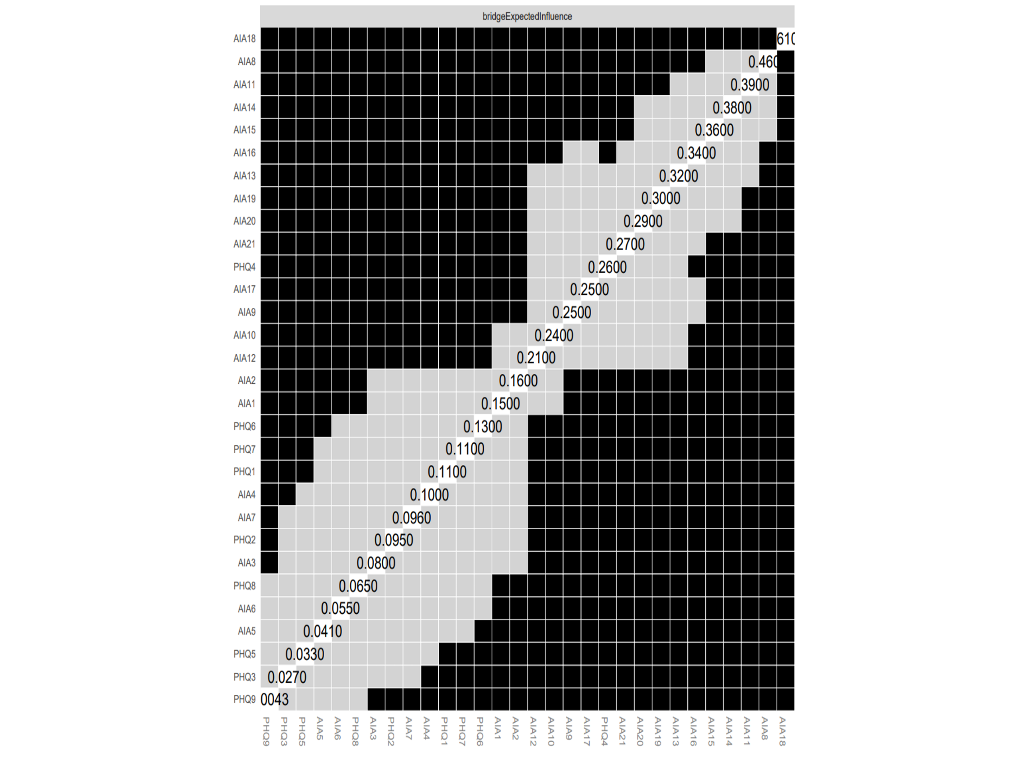

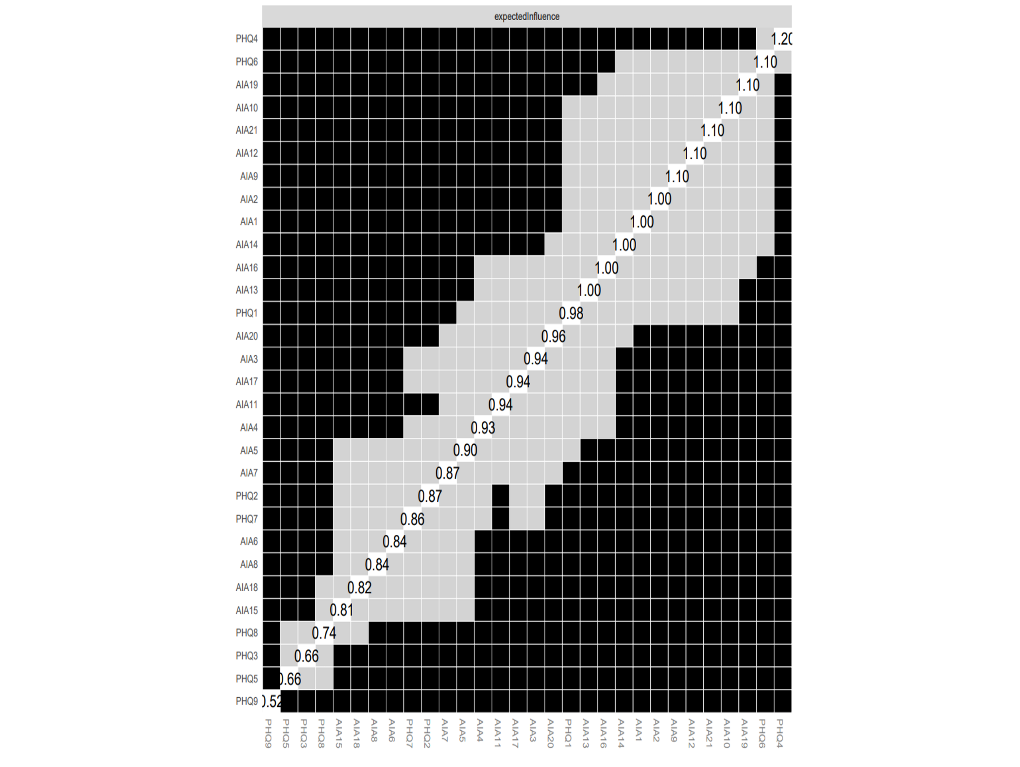


2a 2b

Figure S2. Non-parametric bootstrapping difference test for centrality indices
